# Effects of hunger on mood and affect reactivity to monetary reward in women with obesity – A pilot study

**DOI:** 10.1371/journal.pone.0232813

**Published:** 2020-05-19

**Authors:** Mayron Piccolo, Gabriella Milos, Sena Bluemel, Sonja Schumacher, Christoph Müller-Pfeiffer, Michael Fried, Monique Ernst, Chantal Martin-Soelch

**Affiliations:** 1 Unit of Clinical and Health Psychology, University of Fribourg, Fribourg, Switzerland; 2 Department of Consultation-Liaison-Psychiatry and Psychosomatic Medicine, University Hospital Zurich, University of Zurich, Zurich, Switzerland; 3 Division of Gastroenterology and Hepatology, University Hospital of Zurich, Zurich, Switzerland; 4 Zurich Center for Integrative Human Physiology, Zurich, Switzerland; 5 Section on Neurobiology of Fear and Anxiety, National Institutes of Mental Health, Bethesda, Maryland, United States America; eCampus University, ITALY

## Abstract

Worldwide, nearly 3 million people die every year because of being overweight or obese. Although obesity is a metabolic disease, behavioral aspects are important in its etiology. Hunger changes the rewarding potential of food in normal-weight controls. In obesity, impairments related to reward processing are present, but it is not clear whether these are due to mental disorders more common among this population. Therefore, in this pilot study, we aimed at investigating whether fasting influence mood reactivity to reward in people with obesity. Women with obesity (n = 11, all mentally healthy) and normal weight controls (n = 17) were compared on a computerized monetary reward task (the wheel of fortune), using self-reports of mood and affect (e.g., PANAS and mood evaluation during the task) as dependent variables. This task was done in 2 satiety conditions, during fasting and after eating. Partially, in line with our expectation of a reduced affect and mood reactivity to monetary reward in participants with obesity accentuated by fasting, our results indicated a significant within-group difference across time (before and after the task), with monetary gains significantly improving positive affect in healthy controls (p>0.001), but not in individuals with obesity (p = 0.32). There were no significant between-group differences in positive affect before (p = 0.328) and after (p = 0.70) the task. In addition, women with obesity, compared to controls, reported more negative affect in general (p < 0.05) and less mood reactivity during the task in response to risky gains (p < 0.001) than healthy controls. The latter was independent of the level of satiety. These preliminary results suggest an impairment in mood reactivity to monetary reward in women with obesity which is not connected to the fasting state. Increasing the reinforcing potential of rewards other than food in obesity may be one target of intervention in order to verify if that could reduce overeating.

## Introduction

Obesity represents an increasing public health concern [1]. Worldwide, nearly 3 million people die every year from being overweight or obese. Its prevalence has nearly doubled in the past 30 years [1], leading to what has been described as a global obesity epidemic [2]. Individuals with obesity often suffer from somatic comorbidities, including diabetes, cardiovascular diseases and cancer, among others [1]. Due to possible social stigmatization and discrimination, depression and other psychological issues are more common among this population in comparison to healthy controls (HC) [3]. Although obesity is a metabolic disease, insights from behavioral research could provide new knowledge to the field.

Obesity has been related to perturbations in reward processing [4]. Enhanced activation patterns in cerebral areas related to reward processing were seen in response to palatable food in patients with obesity (OB) relative to normal-weight controls (HC) during both fasting and after food intake [5]. In contrast, normal-weight individuals exhibited striatal activation in response to high-caloric food only during fasting [6]. Consistent with these findings, a recent questionnaire study reported that ‘feelings of reward and achievement’ (measured daily by a visual analogue scales as women were fasting) increased progressively with greater fasting time in normal-weight women [7]. This suggests that food is rewarding for OB independently of the satiety state, while in HC, reward to highly palatable food is mostly present when fasting.

Hypersensitivity to food rewards has been proposed to underlie the increased food consumption, leading to excessive weight gain in obesity [4]. However, it remains unclear how other types of reward are processed in obesity. This question is important because overeating, one of the main causes for obesity [8], might serve as a compensatory behavior for a diminished response to non-food rewards [4]. According to this model, individuals with obesity would evidence blunted responses to food and non-food rewards.

In that context, studies investigating reactions to non-food rewards in participants with obesity–most research works were conducted using monetary reward–showed inconsistent results [9], and behavioral studies which compared behavioral responses to monetary reward in individuals with obesity compared to a control group have yielded mixed results. For instance, Verdejo-Roman, Fornito, Soriano-Mas, Vilar-Lopez, & Verdejo-Garcia [10] and Verdejo-Roman, Vilar-Lopez, Navas, Soriano-Mas, & Verdejo-Garcia [11] reported that participants with obesity were willing to pay less money for plain food than controls. Meemken, Kube, Wickner, & Horstmann [12], on the other hand, reported no significant difference between participants with obesity and controls using the same task in a different sample. In a study investigating reward-learning using monetary reward as positive reinforcer in a conditioning task, individuals with obesity did not differ from controls in learning. However, when food was used as positive reinforcer, women, and not men, with obesity showed impaired learning [13]. In a replication study, Meemken et al. [12] found however that individuals with obesity learned better than controls when food was used as an outcome. These studies suggest that different responses to monetary rewards can be found between healthy controls and individuals with obesity [10], but that these differences are dependent on the methodology and the samples used.

Studies investigating delay discounting in obesity participants compared to health controls also yield partially contradictory results. On one hand, Amlung et al. [14] found significant results, with people with obesity tending to prefer smaller short-term rewards to larger long-term ones. On the other hand, a recent review of the literature failed to evidence clear changes in delay discounting in obesity [9], showing that results on decision-making in relation to monetary reward is unconclusive in this population. These negative findings might be related to the different methodologies used and uneven study populations [9]. Moreover, these inconsistent results might reflect the presence of psychiatric comorbidities, tested in some studies, but not others. Screening for binge eating disorder (BED) is important because it is a mental disorder with a prevalence of 30% among people with overweight problems and of 2–5% in the general population [15], and research has shown differences in monetary reward processing between people with obesity with and without BED [16].

Little has been investigated in terms of affect and mood reactivity to monetary rewards in obesity. Pasco and collaborators [17] investigated positive (happiness, joy, interest, excitement, contentment, enthusiasm and alertness) and negative (distress, anger, disgust, fear and shame) affect in people with obesity, who showed increased negative affect in comparison with controls, but no difference in positive affect. Stoeckel et al. [18], on the other hand, reported no differences in negative affect between groups. These differences could be related to the fact that, while one study controlled for hunger [18], the other did not [17], and fasting states have shown to alter reward reactivity [6]. It is however unclear whether different feeding states would affect mood responses to reward in participants with obesity; and in general, it is not clear whether mood in individuals with obesity is differentially altered in response to monetary reward. This is important as overeating could be explained as a compensatory mechanism for blunted responses to non-food rewards [4]. We focus here on affect associated to winning or not winning money on a computerized gamble task, and mood responses immediately following win and non-win trials, during fasting and after food intake in mental-disorder-free females with obesity in this pilot study.

It still remains unclear whether money is as rewarding in individuals with OB as in controls. In addition, fasting has been shown to influence mood reactions to rewards [5, 7], but it is not clear whether individuals with OB without associated mental disorders would show distinct responses to reward under fasting and satiety states. To extend the knowledge on monetary reward among this population, we used a computerized task to investigate the rewarding value of money in obesity by measuring affect and mood reactivity to monetary rewards during fasting and after food intake in mental-disorder-free females with obesity in this pilot study. The main aim of this study was therefore to test whether there was a reduced mood resp. affect reactivity to monetary reward in participants with obesity (OB) compared to healthy controls (HC) and how this change in affect would be influenced by starving. Our first hypothesis postulated that winning money would improve positive affect and decrease negative affect, both measured with the Positive and Negative Affect Schedule (PANAS) [19] in HC more than in OB participants during fasting, with no difference between groups after eating (H1). Secondly, we hypothesized that OB participants would show higher negative affect compared to healthy controls (HC) across all timepoints independently of the satiety state (H2). And thirdly, we expected higher negative affect during fasting compared to fed state in both groups, but stronger in OB participants (H3). Because our reward task used different probability conditions and because a previous study by our group indicated that reward-related mood changes can be influenced by probability conditions in participants with an eating disorder, i.e. anorexia [20, 21], we performed exploratory analyses to test how self-reports of mood evaluation during the task would be influenced by winning probabilities in OB compared to HC participants and whether the satiety states would affect the interaction between group and winning probabilities.

## Methods

### Ethics

The study was carried out according to Good Clinical Practice and the Declaration of Helsinki. The study protocol was approved by the Ethics Committee of the Canton Zurich (KEK-ZH-No 2009-0115/1). Written informed consent were provided by every participant.

### Participants

Twenty-eight women participated in this study (17 healthy controls and 11 participants with OB). The including criteria for the group with obesity was a body mass index (BMI) higher than 30 kg/m^2^. For the control group, the BMI had to range between 18.5 and 24.9 kg/m^2^ (see Table 1). Excluding criteria were age <18 years and >60 years, and the presence of mental disorders. The Mini International Neuropsychiatric Interview (M.I.N.I) [22], and the German version of the Structured Interview for Anorexic and Bulimic Disorders [23] were used to exclude patients with a history or presence of mental disorders from the obesity and control groups. The participants with obesity were recruited from the outpatient clinic of the Division of Endocrinology of the University Hospital Zurich and via public announcements. Normal-weight healthy controls were recruited via public announcements. The results for the control group have been published elsewhere in comparison to a group of patients with anorexia nervosa [20]. Data were simultaneously collected with this associated study. Since the recruitment for both studies was done at the same time, the same variables were controlled for both studies.

**Table 1:** Description of the study population.

	HC	OB	t	Sig.
**Participants**	17	11		
**Age [years, mean]**	23 ± 5.1	28 ± 8.4	-2.087	0.177
**BMI [kg/m2, mean]**	21.8 ± 1.7	34.8 ± 4.7	-10.369	0.000 ^a^
**Money won [Swiss Francs, CHF]** ^b^	79.65 ± 13.38	82.72 ± 16.26	-.547	.575

Descriptive data are given as mean ± standard deviation.

^a^ indicates a significant different distribution between groups (Independent Samples T Test, p value < 0.001);

^b^ CHF 1 is approximately equivalent to USD 1.

### The Wheel of Fortune task (WoF)

**Fig 1:**
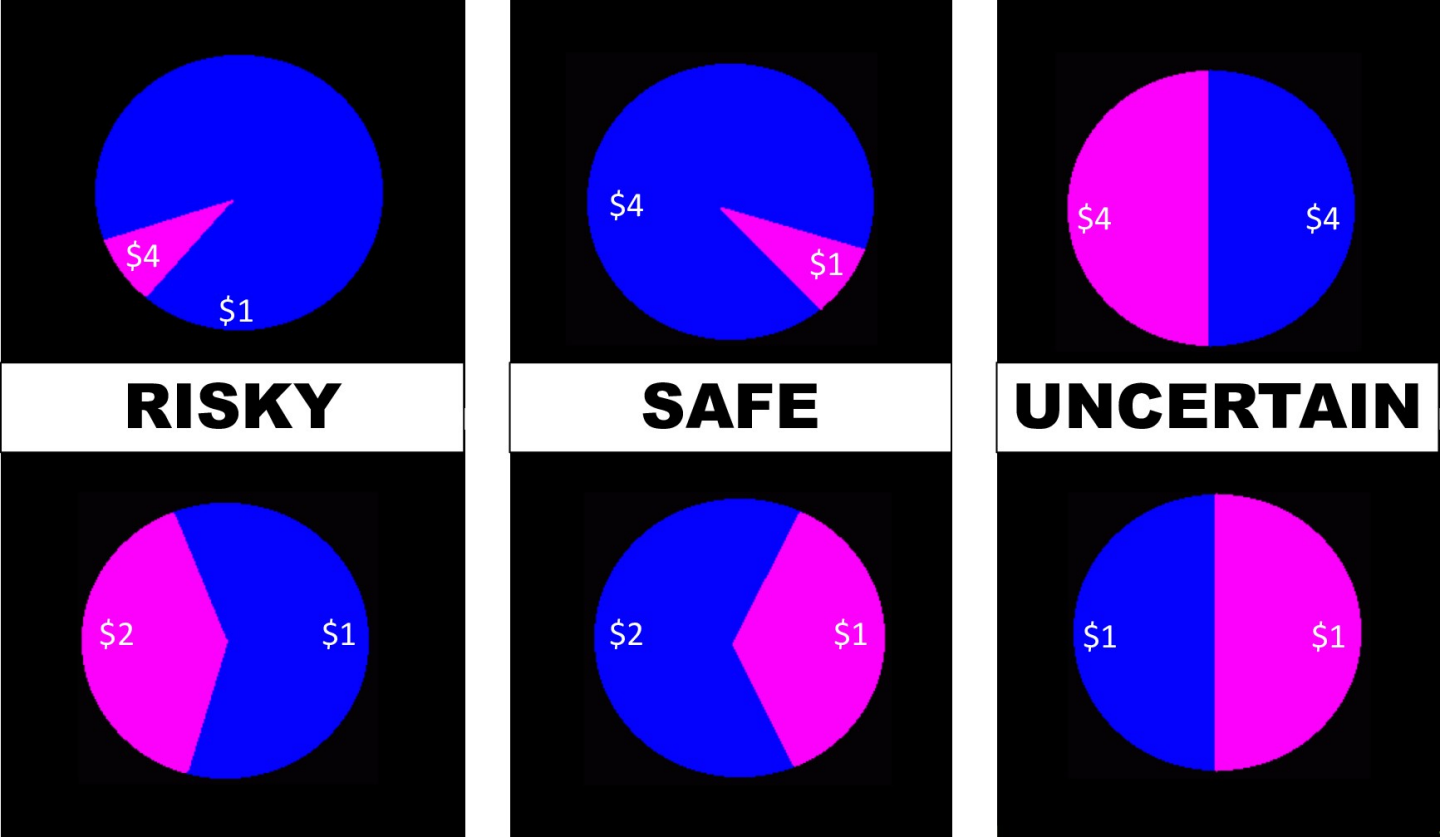
The Wheel of Fortune. The Wheel of Fortune, a computerized task used to measure responses to monetary rewards under risky, safe and uncertain conditions.

The WOF [24] consists of a computerized task involving different winning probabilities. Each trial displays a wheel of fortune. The task comprises three different wheels, based on the probabilities and amounts of win/lose: two 50/50 wheels, one with high and the other with low monetary rewards; two 30/70 wheels, one safe and the other risky; and two 10/90 wheels, one safe and the other risky (Fig 1). Choosing the smallest probability (30 or 10) associated with the higher amount was considered a risky choice, whilst choosing the bigger probability (70 or 90) in association with the higher amount, a safe one. When the option chosen by the participant was the same as the one that had been randomly chosen by the computer, the participants won the chosen amount of money. Otherwise, they did not win any money. After each win/lose feedback, participants were asked to rate their mood in accordance to the previous performance (loss, win) using an emoji visual analogue scale, ranging from 1 (neutral) to 5 (the saddest in the case of loss, or happiest in the case of winning) in response to the question: “how happy/sad do you feel at the moment?”. After a 3-trial familiarization practice (50/50 wheels), participants performed a total of 62 trials during two runs of 31 trials (11 10/90 wheels; 8 30/70 wheels; and 12 50/50 wheels) each. The 50/50 wheels were included because they reflect decision-making during maximum uncertainty [20, 21, 24]. Prizes were as follows: for the 10/90 risky wheels, 10 represented a chance of winning CHF 4, and 90 a chance of winning CHF 1. For the 10/90 safe wheels, 10 represented a chance of winning CHF 1, while 90, CHF 4. For the 30/70 risky wheels, 30 represented the chance of getting CHF 1, while 70, CHF 0.50. For the 30/70 safe wheels, 30 represented the chance of winning CHF 0.50, and 70, CHF 1. The 50/50 wheels included either a high reward (CHF 4), or a low reward (CHF 1) for both wheels. The experiment took between 20- and 30-min, and at the end of the experiment, the participants were given the amount of money won and were compensated for their transportation costs.

### Procedure

The data were collected during a gastric motility study published recently, where further details of the study procedures can be found [25]. In brief, participants fasted for 8 hours prior to the experimentation. After that period, participants were asked to complete the Positive and Negative Affect Schedule (PANAS) [19], a 20-item self-reported measure of positive (enthusiastic, interested, determined, excited, inspired, alert, active, strong, proud, and attentive) and negative affect (scared, afraid, upset, distressed, jittery, nervous, ashamed, guilty, irritable, and hostile) (T1); the WoF, to measure reward-related responses; followed by a second round of the PANAS (T2), i.e., affect was evaluated at two different timepoints: before (T1) and after (T2) the task. T1, WoF and T2 was considered a test round. Participants then ate a standardized meal (430 kcal, 21% fat, 63% carbohydrate, 16% protein) [25]. After 4 hours (fed state), a second muffin was ingested, and a second test round was performed. The intervals were also related to Bluemel and collaborators’ [25] testing. At each state (fasting and fed), hunger was also measured by a previously used procedure [26]. At each time point, participants were asked to rate their perceived hunger using visual analogue scale from *zero* (not hungry) to *one hundred* (very hungry).

### Data analysis and statistics

To increase statistical power, the general linear model, a type of multilevel analyses, was used to perform our data analyses. This type of analyses allows for the use of each trial rather than the use of general means and was used in previous publications of our group with the wheel of fortune task and with similar experimental settings [20, 21, 27, 28]. All analyses were performed using IBM SPSS Statistics 25 (IBM Corp. Armonk, NY, USA).

We ran a total of 5 models. The first two models used the PANAS [19] scores as dependent variable (one with positive affects and one with negative affects as dependent variables) and group (OB versus HC participants), time (before and after the task, i.e. T1 versus T2) and satiety state (fasting versus fed state) as independent variables (fixed factors) to test hypotheses H1 to H3. More specifically, we expected a significant threefold interaction between group x time x satiety state for both positive and negative affects to test H1. To test H2, we expected a significant main effect of group for the negative affects. And finally, to test H3, we expected a twofold interaction group x satiety state for negative affects. The third and fourth models used self-reported mood evaluations during the task as dependent variables and group, satiety state, and decision category (50/50 high, 50/50 low, 30/70 risky, 30/70 safe, 10/90 risky, and 10/90 safe) as independent variables (fixed factors). Model 3 was performed using only winning trials, and model 4 used only not winning trials. For these exploratory analyses, we were interested in the threefold interaction between group x satiety state x probability conditions. The last model (model 5) used self-report of hunger as dependent variable and satiety states as independent variable (independent factor) to control whether the experimental induction of starving resp. satiety had worked. For all models, a heterogeneous first-order autoregressive covariance structure was used for the repeated observations (sessions and trial blocks). In all models, subjects were treated as a random effect. To best account for correlations between repeated measurements, all models were optimized by the covariance type for the repeated observations which produced the lowest Akaike’s Information Criterion [29]. Bonferroni corrections were applied to all comparisons, and the reported p values are those that survived to the corrections at a significance level of p<0.05 after Bonferroni corrections.

## Results

### Hypothesis 1

**Fig 2:**
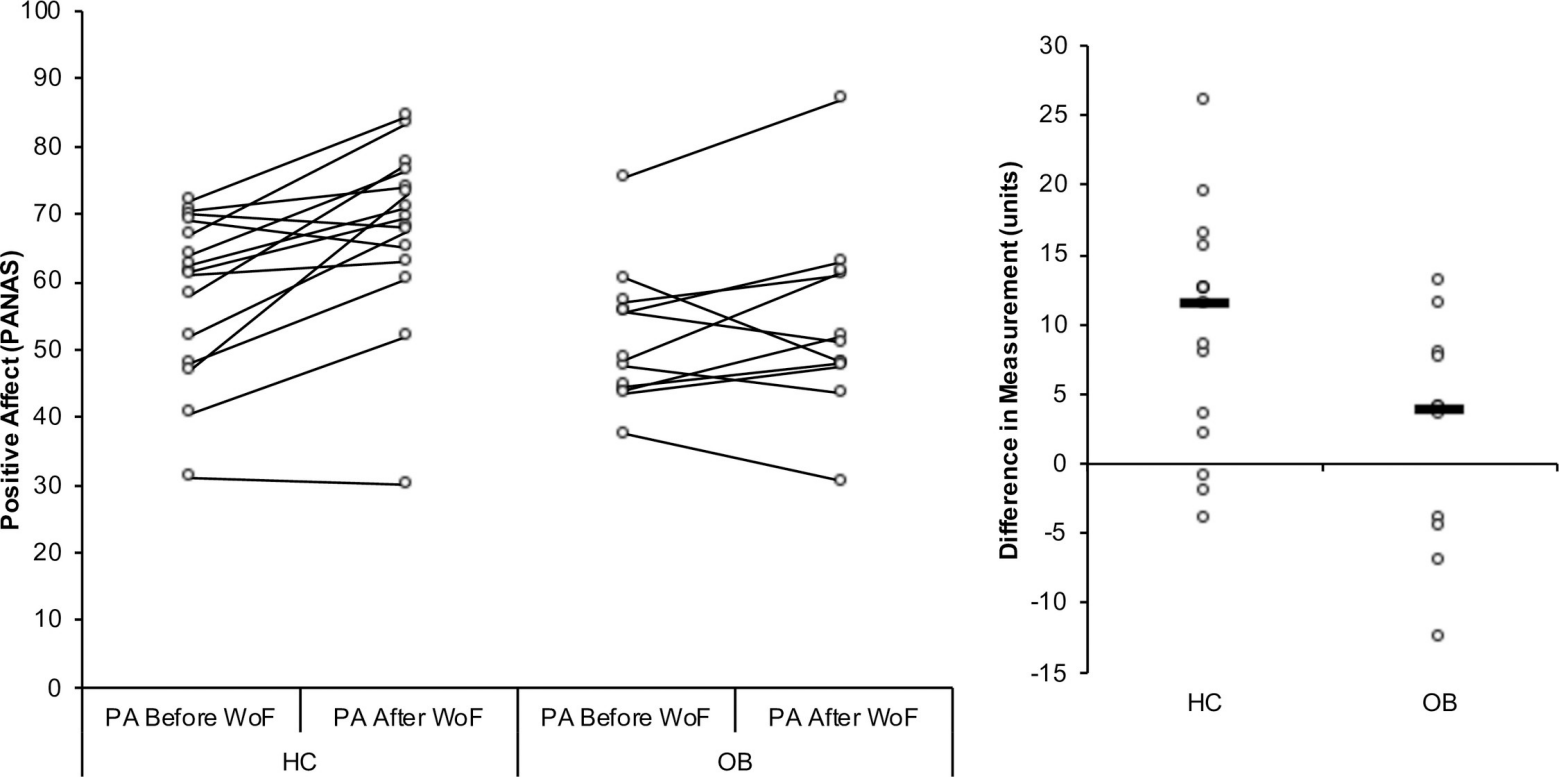
Affect ratings. Affect rating as measured by the PANAS across groups. Overall, OB showed more negative affect than HC (*p < 0.05) regardless the state. Only HC showed increased positive affect after the reward task (T2) in comparison to T1 (**p < 0.05). There was no significant difference for OB.

Positive and negative affect was measured by using the PANAS. There was a significant main effect of time (T1, T2) (F_(1, 35.2)_ = 14.69, p < 0.001) and of an interaction between group x time (F_(1, 35.2)_ = 5.06, p < 0.05) for positive affect (means (M) and standard errors (SE) in Fig 2). In general, more positive affect was reported after the task (M = 60.4, SE = 2.8) than before it (M = 54.9, SE = 2.2) by all participants. Partially confirming H1, the group x time interaction (Fig 2) revealed that HC’s positive affect improved after winning money in the task (T1: M = 57.2, SE = 2.7; T2: M = 65.9, SE = 3.5), while no improvement was seen for OB (T1: M = 52.7, SE = 3.5; T2: M = 55.0, SE = 4.4). No difference was seen between groups before (p = 0.328) or after (p = 0.70) the task. No difference between groups or state (fasting vs fed) was observed for positive affect (group p = 0.133; state p = 0.741).

### Hypothesis 2

For negative affect (Fig 2), a main effect was seen for group (F_(1, 26.1)_ = 6.46, p < 0.05). Confirming hypothesis 2, OB reported more negative affect (M = 12.7, SE = 2.2) than HC (M = 5.6, SE = 1.7) overall. There was no effect of group x time for negative affect (F_(1, 38.922)_ = 0.331, p = 0.568).

### Hypothesis 3

A main effect was also seen for state (F_(1, 28.2)_ = 6.64, p < 0.05) in negative affect. Partially confirming hypothesis 3, more negative affect was reported during fasting (M = 10.1, SE = 1.4) compared to fed (M = 8.2, E = 1.5) across groups, The interaction group x state revealed a trend (p = 0.072), with HC but not OB showing a trend to diminished negative affect after eating (fasting: HC, M = 7.25, SE = 1.799; OB, M = 13.027, SE = 2.237; fed: HC, M = 3.997, SE = 1.829; OB, M = 12.511, SE = 2.299).

### Hypothesis 4

Responses to reward were measured on the Wheel of Fortune task [20]. Exploratory analyses were run on mood in response to reward was assessed after win and no-win trials. Groups did not differ in amount of money won (Table 1).

#### Mood after win trials

**Fig 3:**
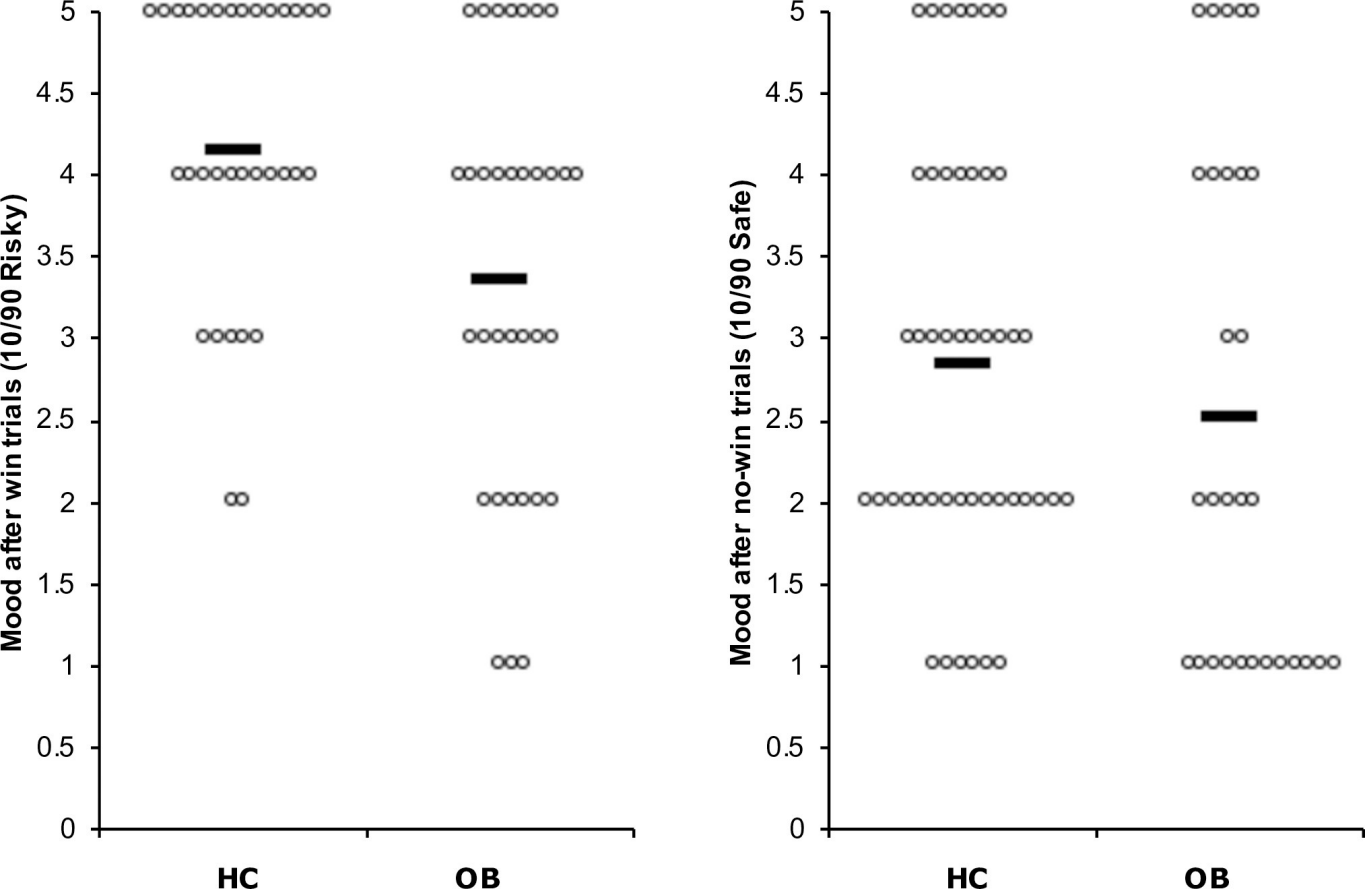
Mood ratings. Mood reactivity to winning and not winning in the 10/90 conditions. HC showed higher positive mood ratings in comparison to OB in the 10/90 risky conditions, that involved a high and more unlikely monetary reward (p < 0.05).

Significant main effects of decision category (F_(5, 1532.9)_ = 74.24, p < 0.001) as well as interactions of group x decision category (F_(5, 1532.9)_ = 4.19, p < 0.001) and satiety state x decision category (F_(5, 1551.6)_ = 2.28, p < 0.05) were found for positive mood after win trials. Regardless of the satiety state, subjects reported higher positive mood after winning in the conditions that involved higher and riskier rewards in comparison to those involving lower and safer rewards. The analysis of group x decision category interaction showed that when the highest and riskiest category was involved, HC reported more positive mood than OB after winning (Fig 3). Satiety state x decision category interaction analysis revealed that across groups, more positive mood was reported during fasting after 10/90 risky. Means and standard errors for positive mood after winning are shown in S1 Table.

#### Mood after no-win trials

Significant main effects of decision category (F_(5, 1444.6)_ = 10.41, p < 0.001) and group x decision category interaction (F_(5, 1444.6)_ = 2.34, p < 0.05) were found for negative mood after no-win trials. Regardless of the satiety state, more negative mood was reported after not winning in the categories that involved high rewards of equal possibility and those involving the safest decisions in comparison to most categories (S1 Table). Group x decision category interaction analysis revealed that these differences were mostly among HC, with no significant difference found for OB.

### Self-reported hunger

Hunger was assessed using a specific scale [26]. Significant effects of state (F_(1, 68.8)_ = 37.62, p < 0.001) as well as an interaction of group x state (F_(1, 68.8)_ = 7.03, p < 0.01) were observed for hunger. Across groups, subjects reported more hunger in the fasted state (M = 48.3, SE = 5.0) than in the fed state (M = 19.9, SE = 4.2). The analysis of the interaction of group x state showed that both groups reported higher levels of hunger in the fasted state (HC: M = 54.6, SE = 6.3; OB: M = 42.0, SE = 7.8) compared to fed (HC: M = 13.9, SE = 5.2; OB: M = 25.8, SE = 6.7, p values < 0.05). The difference between feeding states was significantly higher for HC (Δ = -40 points) in comparison to OB (Δ = -16 points).

## Discussion

Here we present preliminary data of the effect of feeding states on mood reactivity to monetary reward in individuals with obesity in comparison to normal-weight controls. According to our hypotheses, women with obesity showed higher negative affect than normal weight controls, and this was regardless of the satiety state. Although positive affect was improved after the task in general, this effect was stronger in healthy controls, suggesting a poorer value of monetary reward to improve affect among women with obesity. Further exploratory analysis on mood revealed less mood reactivity in obesity in response to the different winning/losing possibilities regardless of the satiety state. Finally, women with obesity showed less positive mood after winning the highest and riskiest reward, and, accordingly, less negative mood after losing the highest and safest reward than normal weight controls.

With regard to the changes in affect, reward had a significant effect of increasing positive affect mostly in healthy controls, independently of the satiety state. On the other hand, the task did not decrease negative affect. Recently, it has been reported that winning money at a monetary task increased positive affect in healthy women [20]. Our results add to the literature suggesting money is not as rewarding for people with obesity as it is for health controls. In line with that, neuroimaging data involving a monetary reward task revealed decreased brain response in reward anticipation among people with obesity [16]. Also, reaction times to low monetary rewards were slower in mental-disorder-free individuals with obesity, suggesting reduced reward sensitivity to money [11]. These findings are in accordance to what would be expected in relation to dopamine availability and striatal activation [30] in the study population [31, 32]. Dopamine availability has been linked to positive affect [30, 33], and is reported to be altered in individuals with obesity [34]. In line with blunted neural responses to reward, negative emotions and other psychological issues are more common among obese individuals relative to healthy controls [3]. Dixon, Dixon and O'Brien [35] reported that the risk of depression, for instance, is higher among obese women. Another interesting fact reported in their research was that this risk decreased with weight loss, strengthening the relation between weight and psychological factors.

Our exploratory analysis revealed that mood reactivity to winning/not winning monetary rewards was not as strong in women with obesity compared to lean women. In healthy women, positive mood was higher when winning a risky than predictable reward, while negative mood was stronger when incurring a predictable than risky loss, which is consistent with previous work [20]. Healthy women showed stronger mood reactivity to winning unpredictable and losing predictable rewards (10/90 conditions), but this pattern was not seen in obesity. To the best of our knowledge, mood reactivity to winning or losing expected vs unexpected monetary rewards has not been previously examined in individuals with obesity. Our results suggest that individuals with obesity would show a diminished capacity for experiencing monetary rewards as positive. Accordingly, Kube and collaborators [36] showed that, neural responses to a monetary task did not differentiate between loss or gain in individuals with obesity, suggesting that reward-related dysfunction also pertains to money and not only to food in this population.

This pilot study showed preliminary data demonstrating that food does not influence the perception of monetary reward in women with obesity, and that winning money does not improve affect in obesity while it does in normal-weight controls. Although the sample size was small, and generalizability of these results might be limited, mixed-model analyses were used, which made it possible to maximize the degrees of freedom by using every trial performed by every subject, and not simply the means of all participants in a group, therefore increasing statistical power. However, small sample sizes may overestimate effects, therefore, future studies should replicate this procedure in larger samples, especially for the affect responses, which represent more an exploratory analysis. Another important issue is that visits were not counterbalanced, with fasting always preceding the fed states. Future studies should counterbalance visits, besides testing other types of reward (e.g. social) in the same population. Finally, these findings cannot be generalized to males, since exclusively women were tested.

To the best of our knowledge, this is the first investigation of mood reactivity in obesity after risky vs safe monetary reward winning, before and after food intake. Our results suggest that eating may not modulate mood reactivity to monetary rewards. Furthermore, our preliminary data suggests blunted mood reactivity to monetary reward in women with obesity, independently of satiety state. This might be linked to impaired DA reactivity. However, this remains to be elucidated. It is important to consider refining interventions/diet counseling and self-education of patients with obesity in a way to increase the rewarding potential of types of reward other than food (e.g. social, monetary). If overeating may be a compensation for a diminished response to non-food rewards, increasing the reinforcing potential of other types of reward may help reduce this behavior in obesity.

## Supporting information

S1 Table Mood. Mood ratings in response to reward in the Wheel of Fortune in women with obesity (OB) and healthy controls (3) during Fasting and Fed States.
(DOCX)
