# Supplementary material for: Effects of hunger on mood and affect reactivity to monetary reward in women with obesity – A pilot study
Source: PLoS One. 2020 May 19;15(5):e0232813. doi: 10.1371/journal.pone.0232813 (PMC7237012; doi:10.1371/journal.pone.0232813)
Supplement: S1 Table — Mood ratings in response to reward in the Wheel of Fortune in women with obesity (OB) and healthy controls (3) during Fasting and Fed States. (DOCX) [file pone.0232813.s001.docx]

**S1 Table.** Mood ratings in response to reward in the Wheel of Fortune in women with obesity (OB) and healthy controls (3) during Fasting and Fed States.

|  | Session | Decision Category | OB (*n*=11), mean | ± | SE | HC (*n*=17), mean | ± | SE | Fixed Effects | F | p value |
| --- | --- | --- | --- | --- | --- | --- | --- | --- | --- | --- | --- |
|  |  |  |  |  |  |  |  |  |  |  |  |
| Positive Mood | Fasting | 50/50 high | 3.61 | ± | 0.28 | 3.93 | ± | 0.23 | Group | 0.935 | 0.342 |
| (to winning) |  | 50/50 low | 3.05 | ± | 0.28 | 3.24 | ± | 0.23 | Session | 0.465 | 0.497 |
|  |  | 30/70 risky | 3.32 | ± | 0.29 | 3.73 | ± | 0.23 | Decision Category | 74.239 | 0.000 |
|  |  | 30/70 safe | 2.70 | ± | 0.29 | 3.27 | ± | 0.23 | Group * Session | 1.728 | 0.191 |
|  |  | 10/90 risky | 3.48 | ± | 0.33 | 4.37 | ± | 0.28 | Group * Decision Category | 4.187 | 0.001 |
|  |  | 10/90 safe | 2.95 | ± | 0.28 | 3.09 | ± | 0.22 | Session * Decision Category | 2.281 | 0.044 |
|  |  |  |  |  |  |  |  |  |  |  |  |
|  | Fed | 50/50 high | 3.72 | ± | 0.28 | 4.00 | ± | 0.23 | Group * Session * Decision Category | 1.527 | 0.178 |
|  |  | 50/50 low | 3.02 | ± | 0.28 | 3.23 | ± | 0.23 |  |  |  |
|  |  | 30/70 risky | 3.56 | ± | 0.29 | 3.58 | ± | 0.23 |  |  |  |
|  |  | 30/70 safe | 3.08 | ± | 0.29 | 3.15 | ± | 0.23 |  |  |  |
|  |  | 10/90 risky | 2.94 | ± | 0.35 | 3.85 | ± | 0.28 |  |  |  |
|  |  | 10/90 safe | 3.03 | ± | 0.28 | 3.05 | ± | 0.22 |  |  |  |
|  |  |  |  |  |  |  |  |  |  |  |  |
|  | Fasting | 50/50 high | 3.66 | ± | 0.27 | 3.96 | ± | 0.22 |  |  |  |
|  | + Fed | 50/50 low | 3.03 | ± | 0.27 | 3.24 | ± | 0.22 |  |  |  |
|  |  | 30/70 risky | 3.44 | ± | 0.28 | 3.66 | ± | 0.22 |  |  |  |
|  |  | 30/70 safe | 2.89 | ± | 0.28 | 3.21 | ± | 0.22 |  |  |  |
|  |  | 10/90 risky | 3.21 | ± | 0.31 | 4.11 | ± | 0.25 |  |  |  |
|  |  | 10/90 safe | 2.99 | ± | 0.27 | 3.07 | ± | 0.22 |  |  |  |
|  |  |  |  |  |  |  |  |  |  |  |  |
| Negative Mood | Fasting | 50/50 high | 2.39 | ± | 0.31 | 2.94 | ± | 0.25 | Group | 0.418 | 0.524 |
| (to losing) |  | 50/50 low | 2.24 | ± | 0.31 | 2.53 | ± | 0.25 | Session | 0.3624 | 0.548 |
|  |  | 30/70 risky | 2.24 | ± | 0.32 | 2.36 | ± | 0.26 | Decision Category | 10.414 | 0.000 |
|  |  | 30/70 safe | 2.22 | ± | 0.32 | 2.60 | ± | 0.26 | Group * Session | 0.052 | 0.820 |
|  |  | 10/90 risky | 2.27 | ± | 0.31 | 2.46 | ± | 0.25 | Group * Decision Category | 2.341 | 0.040 |
|  |  | 10/90 safe | 2.66 | ± | 0.38 | 2.73 | ± | 0.30 | Session * Decision Category | 0.648 | 0.663 |
|  |  |  |  |  |  |  |  |  |  |  |  |
|  | Fed | 50/50 high | 2.56 | ± | 0.32 | 2.93 | ± | 0.25 | Group * Session * Decision Category | 1.241 | 0.288 |
|  |  | 50/50 low | 2.29 | ± | 0.32 | 2.34 | ± | 0.25 |  |  |  |
|  |  | 30/70 risky | 2.40 | ± | 0.33 | 2.49 | ± | 0.26 |  |  |  |
|  |  | 30/70 safe | 2.52 | ± | 0.33 | 2.52 | ± | 0.26 |  |  |  |
|  |  | 10/90 risky | 2.38 | ± | 0.31 | 2.44 | ± | 0.25 |  |  |  |
|  |  | 10/90 safe | 2.37 | ± | 0.38 | 3.13 | ± | 0.30 |  |  |  |
|  |  |  |  |  |  |  |  |  |  |  |  |
|  | Fasting | 50/50 high | 2.48 | ± | 0.30 | 2.93 | ± | 0.24 |  |  |  |
|  | + Fed | 50/50 low | 2.26 | ± | 0.30 | 2.44 | ± | 0.24 |  |  |  |
|  |  | 30/70 risky | 2.32 | ± | 0.30 | 2.43 | ± | 0.24 |  |  |  |
|  |  | 30/70 safe | 2.37 | ± | 0.31 | 2.56 | ± | 0.24 |  |  |  |
|  |  | 10/90 risky | 2.32 | ± | 0.30 | 2.45 | ± | 0.24 |  |  |  |
|  |  | 10/90 safe | 2.51 | ± | 0.34 | 2.93 | ± | 0.26 |  |  |  |
